# The complete mitochondrial genome of the sharpsnout seabream *Diplodus puntazzo* (Perciformes: Sparidae)

**DOI:** 10.1080/23802359.2020.1775509

**Published:** 2020-06-08

**Authors:** Marina Ceruso, Iolanda Venuti, David Osca, Luigi Caputi, Aniello Anastasio, Fabio Crocetta, Paolo Sordino, Tiziana Pepe

**Affiliations:** a Department of Veterinary Medicine and Animal Production, University “Federico II”, Naples, Italy; b Integrative Marine Ecology, Stazione Zoologica Anton Dohrn, Naples, Italy; c Biology and Evolution of Marine Organisms, Stazione Zoologica Anton Dohrn, Naples, Italy

**Keywords:** Mitogenomics, gene organization, base composition, demersal fishes, phylogenetic relationships

## Abstract

The sharpsnout seabream *Diplodus puntazzo* Walbaum, 1792 is a target species of small-scale fishery activities and is cage-cultured for human consumption. Nonetheless, genetic information on this species is limited. We here first sequence its complete mitochondrial genome. The sequence is composed of 16,638 base pairs, accounting for 13 protein-coding genes, 2 rRNA genes, 22 tRNA genes, and 2 non-coding regions (D-loop and L-origin). The overall nucleotide composition is: 27.4% A, 28.9% C, 26.9% T, and 16.8% G. Maximum likelihood analyses placed *D. puntazzo* close to *Acanthopagrus* and some *Pagellus* species.

The sharpsnout seabream *Diplodus puntazzo* Walbaum, 1792 is a coastal and gregarious demersal fish of the family Sparidae, distributed in the eastern Atlantic Ocean and the Mediterranean Sea (e.g. Vinagre et al. 2010). It reaches ∼60 cm in length and exceeds 2–3 kilograms in weight (Pavlidis and Mylonas, 2011), thus representing a target species of small-scale fishery activities and being cage-cultured for human consumption in the Mediterranean basin (Favaloro et al. 2002; Chaouch et al. 2013). Notwithstanding its commercial importance, genetic information on this species is still limited to specific mitochondrial regions (*COI*, *cytb*, D-loop) (e.g. Bargelloni et al. 2005). We fill this gap by sequencing its complete mitochondrial genome (GenBank MT319027). A specimen of *D. puntazzo* was caught in the Gulf of Pozzuoli (Naples, Tyrrhenian Sea, Mediterranean Sea, 40°48′23.3″N, 14°07′08.1″E), identified based on morphological features by one of us (F. Crocetta) and subsequently deposited in the Darwin Dohrn Museum of the Stazione Zoologica Anton Dohrn of Naples with the code number SZN-OST-0001. Mitochondrial DNA was extracted from dorsal fin tissue (Mascolo, Ceruso, Sordino et al. 2019). The assembled and annotated mitogenome was obtained by high-throughput sequencing of enriched mitochondrial DNA with Illumina HiSeq 2500 System (Illumina, San Diego, CA, USA) and bioinformatic analyses were done at Bio-Fab Research (Rome, Italy). The mitogenome is 16,638 base pairs (bp) long, containing 13 protein-coding genes, 2 ribosomal RNA genes (12S and 16S), 22 transfer RNA genes (*tRNA*), and 2 non-coding regions (D-loop and L-origin). Mitochondrial structure and gene organization are in agreement with typical vertebrate mitogenomes (Wang et al. 2008). The majority of mitochondrial genes were encoded on the heavy strand, with the *NADH dehydrogenase subunit 6* (*ND6*) and eight *tRNA* genes [*Gln*, *Ala*, *Asn*, *Cys*, *Tyr*, *Ser (UCN)*, *Glu*, *Pro*] being encoded on the light strand. Base composition is 27.4% A, 28.9% C, 26.9% T, and 16.8% G, similar to that observed in other species of the same family (Ceruso, Mascolo, Palma et al. 2018; Ceruso, Mascolo, Lowe et al. 2018; Mascolo et al. 2018a, 2018b; Mascolo, Ceruso, Chirollo 2019). All protein-coding genes started with an ATG start codon except *COI* and *ND6*, that started with GTG and CTA, respectively. Stop codons were of 5 types, i.e. TAA (*ND1*, *ATP6*, *ND4L*), AGG (*COI*), T (*COII*, *ND3*, *ND4*, *ND6*, *CYTB*), TAG (*ATP8*, *ND5*), and TA (*ND2*, *COIII*). The 12S and 16S rRNA genes were located between the *tRNA^Phe^* (GAA) and *tRNA^Leu^* (TAA) genes and were separated by the *tRNA^Val^* gene as in other vertebrates (Li et al. 2016). The 22 *tRNA* genes vary from 66 to 74 bp in length. The control region (968 bp) is located between *tRNA^Pro^* (TGG) and *tRNA^Phe^* (GAA). The non-coding region (L-strand origin of replication) is 36 bp long and is located between *tRNA^Asn^* (GTT) and *tRNA^Cys^* (GCA).

The phylogenetic position of *D. puntazzo* was investigated performing maximum likelihood (ML) analyses using the RAxML-NG software (Kozlov et al. 2019) (Figure 1). The resultant phylogeny places *D. puntazzo* close to *Acanthopagrus schlegelii* and *Acanthopagrus latus*, with these three species constituting the sister group of *Pagellus bogaraveo* and *Pagellus acarne*. The present study informs on phylogenetic relationships within Sparidae and may provide a baseline for future molecular studies on *D. puntazzo*.

**Figure 1.**
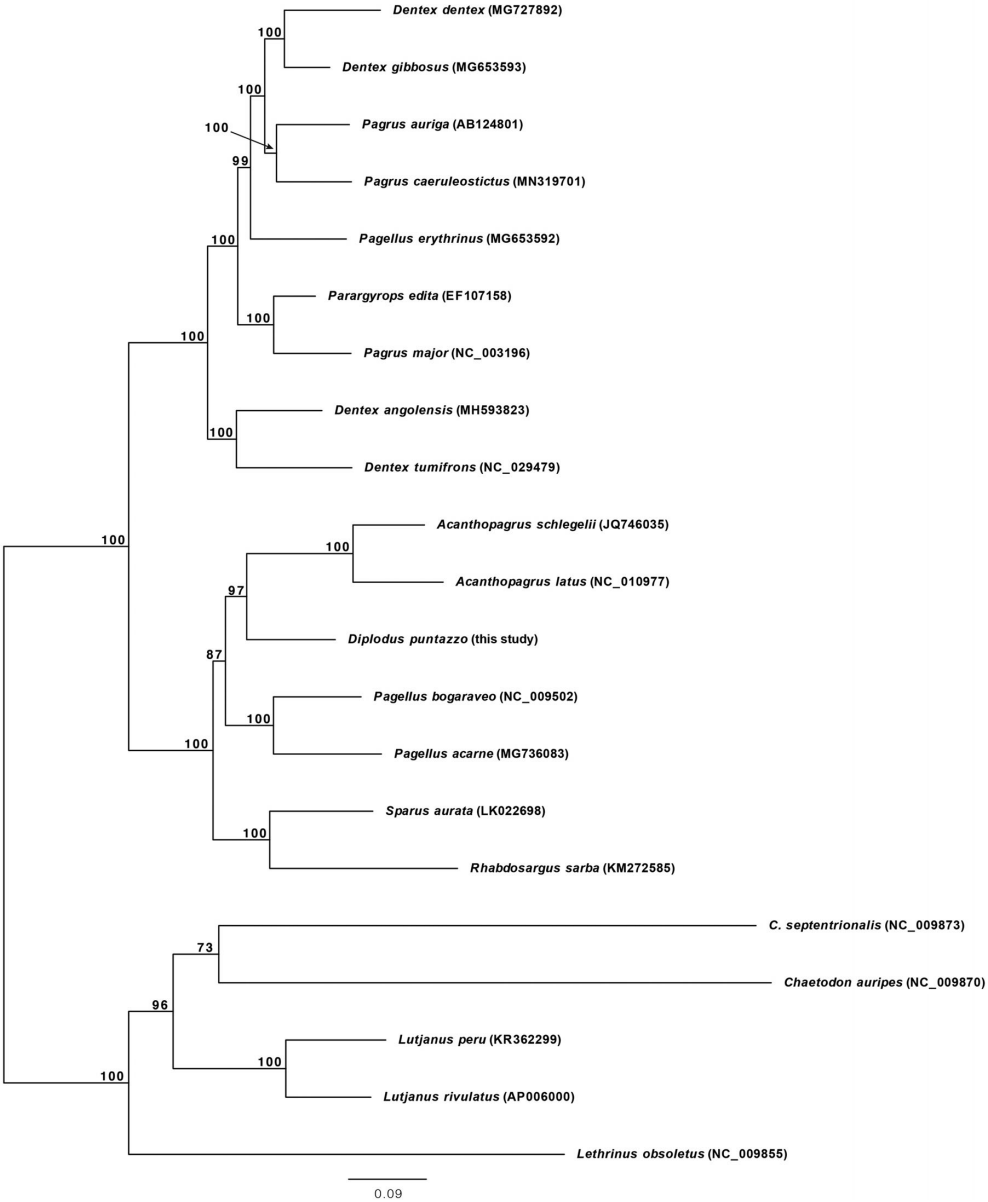
Phylogenetic relationships in the family Sparidae based on the mtDNA sequences available in GenBank and that of *Diplodus puntazzo* reported here (*Acanthopagrus latus* NC_010977, *Acanthopagrus schlegelii* JQ746035, *Dentex angolensis* MH593823, *Dentex dentex* MG727892, *Dentex gibbosus* MG653593, *Dentex tumifrons* NC_029479, *Pagellus acarne* MG736083, *Pagellus bogaraveo* NC_009502, *Pagellus erythrinus* MG653592, *Pagrus auriga* AB124801, *Pagrus caeruleostictus* MN319701, *Pagrus major* NC_003196, *Parargyrops edita* EF107158, *Rhabdosargus sarba* KM272585, *Sparus aurata* LK022698). Five outgroup species (*Chaetodon auripes* NC_009870, *Chaetodontoplus septentrionalis* NC_009873, *Lethrinus obsoletus* NC_009855, *Lutjanus peru* KR362299 and *Lutjanus rivulatus* AP006000) were selected. Maximum likelihood method was used with an automatic bootstrapping cutoff of 0.01.

## Data Availability

The data that support the findings of this study are openly available in Genbank at https://www.ncbi.nlm.nih.gov/nuccore/MT319027, reference accession number MT319027.1.
